# Time Efficiency and Ergonomic Assessment of a Robotic Wheelchair Transfer System

**DOI:** 10.3390/s24237558

**Published:** 2024-11-27

**Authors:** Shantanu A. Satpute, Kaylee J. Uribe, Oluwatofunmi O. Olaore, Minori Iizuka, Ian C. McCumber Gandara, William J. Schoy, Rutuja A. Kulkarni, Rosemarie Cooper, Alicia M. Koontz, Owen Flaugh, Rory A. Cooper

**Affiliations:** 1 Human Engineering Research Laboratories, Department of VA Pittsburgh Healthcare System, School of Health and Rehabilitation Sciences, University of Pittsburgh, Pittsburgh, PA 15206, USA; shs220@pitt.edu (S.A.S.); kju11@pitt.edu (K.J.U.); oreolaore@gmail.com (O.O.O.); minori.iizuka@mail.mcgill.ca (M.I.); ian.mccumbergandara@westpoint.edu (I.C.M.G.); wjs43@pitt.edu (W.J.S.IV); ruk20@pitt.edu (R.A.K.); cooperrm@pitt.edu (R.C.); akoontz@pitt.edu (A.M.K.); owf2@pitt.edu (O.F.); 2 Department of Bioengineering, Swanson School of Engineering, University of Pittsburgh, Pittsburgh, PA 15261, USA; 3 Department of Electrical Engineering and Computer Science, United States Military Academy, West Point, NY 10996, USA

**Keywords:** disability, motion capture, inertial measurement units, biomechanics, caregivers, nurses, back pain, musculoskeletal injuries, assistive technology

## Abstract

**Background**: Caregivers experience high rates of occupational injuries, especially during wheelchair transfers, which often result in back pain and musculoskeletal disorders due to the physical demands of lifting and repositioning. While mechanical floor lifts, the current standard, reduce back strain, they are time-consuming and require handling techniques that subject caregivers to prolonged and repeated non-neutral trunk postures, increasing the risk of long-term back injuries. **Aims**: The aim was to assess the time efficiency and ergonomics of the powered personal transfer system (PPTS), a robotic transfer device designed for bed-to/from-wheelchair transfers. **Methods**: We evaluated transfers with the PPTS and mechanical lift with eight able-bodied participants who assisted with transfers between a bed and a wheelchair. Inertial measurement units (IMUs) were placed on participants to track their motion and assess trunk joint angles during transfers. **Results**: The PPTS significantly reduced the transfer time (144.31 s vs. 525.82 s, *p* < 0.001) and required significantly less range of motion for trunk flexion (*p* < 0.001), lateral bending (*p* = 0.008), and axial rotation (*p* = 0.001), all of which have been associated with back injuries. Additionally, the PPTS significantly reduced the time caregivers spent in non-neutral trunk postures, potentially lowering injury risks. **Conclusions**: These findings suggest that the PPTS improves transfer efficiency and caregiver safety, offering a promising alternative to the current standard of care for wheelchair-to/from-bed transfers.

## 1. Introduction

Wheelchair transfers play a crucial role in enhancing the mobility and independence of individuals using wheelchairs (WCs). However, the process of transferring between a WC to/from other surfaces can be physically demanding and pose inherent risks to both WC users and the caregivers [1,2]. In the U.S., about 5.5 million people, including 250,000 veterans, rely on WCs for mobility, often requiring caregiver assistance for daily activities such as transfers [3]. Assisted transfers, while critical for mobility, tend to increase the risk of injury for both parties.

The Bureau of Labor Statistics reports caregivers having the highest rate of nonfatal occupational injuries, exceeding those in construction or other heavy-duty jobs [4,5]. Such a high incidence of occupational injury is a result of frequent involvement in assisting with transfers, a task that often requires repeated handling and repositioning [4,5,6,7,8]. The healthcare sector, one of the largest and fastest-growing industries in the U.S., reports high rates of overexertion injuries, particularly affecting the back, classified as musculoskeletal disorders (MSDs). These injuries are strongly linked to job tasks requiring forceful exertion, repetitive movements, and awkward postures [9]. Manual transfers, which involve lifting and moving the care recipient, often exceed safe limits for back forces, exposing caregivers to increased biomechanical risks, including compressive and shear forces on the lumbar spine [2,10,11]. During manual transfers, caregivers spend approximately 25% of their time in a trunk flexion greater than 30 degrees, leading to repetitive awkward bending and rotation of the trunk [6,7,12]. This postural strain, including lateral bending and axial trunk rotation, is a key contributor to discomfort and musculoskeletal injuries, with 51% of caregivers citing it as a source of pain [5,7]. Musculoskeletal injuries rank third in total costs for occupational injuries, amounting to USD 195.4 billion annually [13]. Transfers and patient lifts are the most frequent causes of back injuries among healthcare workers, with financial costs from injuries exceeding USD 900 million for registered nurses, USD 40 million for licensed practical nurses, and USD 2.2 billion for aides and attendants [14]. Prolonged periods of awkward trunk posturing heighten the risk of low back pain, which affects 50% of healthcare workers annually and 80% over the course of their careers [4,5].

Several transfer devices have been developed and employed to mitigate risks with manual transfers [15]. Sliding boards reduce forces to an extent but remain physically demanding and do not substantially diminish the risk of injury [16]. Mechanical floor and ceiling lifts have been proven effective in reducing back forces, movements, and biomechanical stress. A recent literature review of biomechanical studies on transfers for caregivers highlighted that this method is preferred for minimizing the incidence of injuries and is widely adopted as the standard of care [16,17].

While much safer than manual transfers, mechanical lifts are often inconvenient and time inefficient. A study of 27 nurses in a long-term care facility found that they were twice as likely to use manual lifting for wheelchair transfers. This was primarily due to ceiling- and floor-based mechanical lifts taking 3.5 to 8.3 times longer to operate, making them less practical for frequent use [18]. Mechanical lifts require caregivers to position a sling under the care recipient, involving lifting, repositioning, and sliding, which can still be physically demanding [19].

The powered personal transfer system (PPTS) aims to improve the safety and efficiency of wheelchair transfers by potentially reducing the physical strain associated with mechanical lifts [20,21]. This robotic wheelchair transfer system, which includes a hospital bed and a powered wheelchair, is designed to eliminate the need for manual lifting and sling positioning, which may reduce the physical demands on caregivers. Through a touchscreen interface, caregivers can operate the system by pressing and holding a button to coordinate the movement of the bed and the wheelchair. This automation may allow caregivers to focus on guiding the person being transferred while the PPTS manages the rest, potentially enhancing safety and comfort for the individual and reducing caregiver injury. The PPTS offers a no-lift solution for bed-to-wheelchair transfers, which are among the most physically demanding [22].

Previous research on the PPTS has demonstrated its potential benefits for users and caregivers. Initial focus group studies with 18 wheelchair users and 18 caregivers confirmed the feasibility and practicality of the PPTS [20]. Furthermore, an evaluation involving 15 caregivers using the PPTS to transfer an anthropometric life-sized dummy revealed lower workload demands and greater perceived safety and efficiency compared with current transfer methods [21]. Despite these promising results, previous studies have relied primarily on subjective usability assessments and lack objective measures of the PPTS’s ergonomic advantages relative to current standard practices. This study aimed to address these gaps with the following objectives:

**Objective 1**: Compare the time efficiency of the PPTS with a mechanical floor lift (the current standard of care).

**Hypothesis** **1.** *We hypothesized that the PPTS would enable significantly faster transfers compared with the mechanical floor lift*.

**Hypothesis** **2.** *We hypothesized that transfers with the PPTS would be more consistent (lower variance) in duration compared with the mechanical floor lift, indicating less variability and a more predictable transfer process*.

**Objective 2**: Objectively determine which transfer device supports more ergonomic posture by quantifying caregiver trunk joint angles during transfers.

**Hypothesis** **3.** *We hypothesized that the PPTS would require lower trunk flexion/extension (bending forward/backwards), lateral bending (bending sideways), and axial rotation (twisting) compared with the mechanical floor lift*.

Caregivers may be repeatedly exposed to these risky postures during a typical eight- to twelve-hour shift, which can increase the risk of back injuries and discomfort. Specifically, trunk flexion [23], lateral bending, and axial rotation [24,25,26] exceeding 20° are known risk factors for back injuries [27,28,29]. Additionally, the duration spent in non-neutral trunk postures is associated with back pain [23,29]. We aimed to assess the amount of time spent in these non-ergonomic postures.

**Hypothesis** **4.** *We hypothesized that transfers using the PPTS would require significantly lower durations of trunk joint angles above 20°, compared with the mechanical floor lift, thereby indicating a reduction in the potential risk of back injuries and discomfort*.

## 2. Methods

### 2.1. Participants

The study was conducted in accordance with the guidelines of the Declaration of Helsinki and approved by the Institutional Review Board at the University of Pittsburgh (STUDY24060020, approved on 10 July 2024). All participants provided informed consent by signing a consent form prior to participating in the study.

The sample size for this study was determined based on the prior literature on transfer time and trunk flexion during patient transfers. Specifically, we based our calculations on two key parameters: time taken to complete transfers and trunk flexion angles. Prior research has reported an average transfer time of 289.4 ± 48.4 s for transfers using mechanical floor lift devices, such as the Hoyer lift [30]. Initial trials with the PPTS indicated an average transfer time of approximately 150 ± 10 s, accounting for variations in participant height. This difference corresponded to a Cohen’s d effect size of 3.24. Using G*Power software, a sample size of 4 participants was calculated to achieve 80% power for a paired t-test with α = 0.05. In terms of trunk flexion, a study on nurses in long-term care facilities found that the maximum trunk flexion during patient transfers reached 80° [18]. In comparison, our preliminary trials with the PPTS showed an average trunk flexion of 25° (SD = ±10°). This difference yielded a Cohen’s d effect size of 1.88, suggesting a sample size of 6 participants would be sufficient for statistical significance. We aimed to recruit 8 participants to be conservative in our estimations. This sample size was consistent with the sample sizes used in other biomechanical studies comparing assistive technologies [31,32].

Participants were required to be at least 18 years old. Exclusion criteria included pregnancy, people with a physical disability, or those experiencing or having recently experienced musculoskeletal pain that would impede their ability to assist with transfers. Participants were not required to have prior experience with patient transfers. While this may represent a potential limitation, the general population who serve as caregivers lack formal training in transfer techniques. Including participants without transfer experience accurately reflects the context of family caregiving [33,34].

To simulate the role of the wheelchair user during transfers, a member of the research team (weight = 73 kg, height = 173 cm) served as a surrogate WC user. This decision was made to avoid placing an undue physical burden on actual wheelchair users as the study required participants to perform multiple transfers (eight in total) over a short two-hour period. Surrogating a research team member as the wheelchair user ensured consistency and reduced the study risk.

### 2.2. Motion Capture

**Figure 1:**
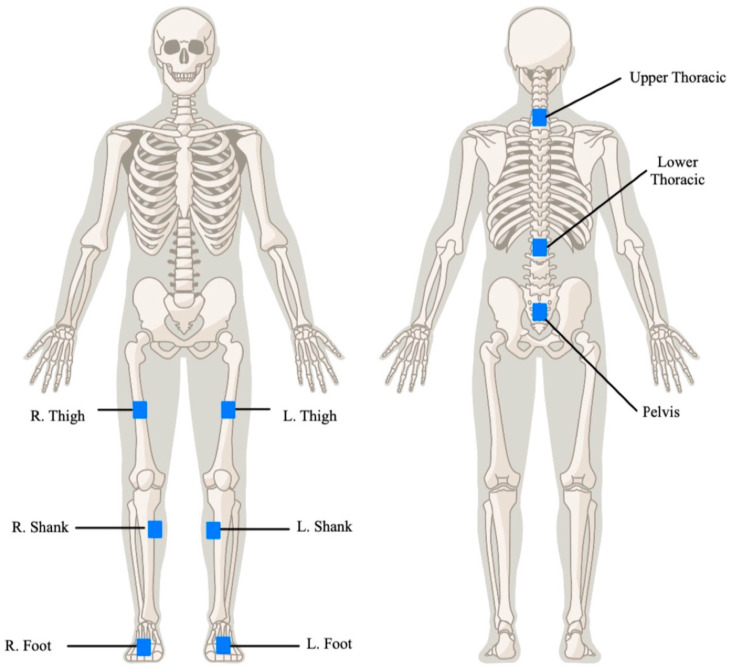
IMU placement for motion capture.

To quantify trunk joint angles during participant-assisted transfers using both the mechanical floor lift and the PPTS, we needed to accurately track participant movement throughout the transfer process. We used Noraxon inertial measurement units (IMUs) to capture motion data. IMUs integrate tri-axial accelerometers, gyroscopes, and magnetometers to compute relative orientation in three-dimensional space. Through sensor fusion, IMUs provide minimal drift orientation data, which can be used to derive joint angles. A systematic review demonstrated that IMUs show a root mean squared error (RMSE) of less than 2.4° when compared with the gold standard (optical motion capture), with intraclass correlation coefficients (ICCs) greater than 0.84, indicating high accuracy and reliability for measuring low back movements [35,36,37]. IMUs have shown excellent validity and reliability in dynamic scenarios, such as those requiring fast trunk movements [36]. Sensor placement followed the Noraxon’s user guide. Figure 1 illustrates the IMU placements.

### 2.3. Training

Participants were trained to use both the PPTS and the mechanical floor lift. The training began with participants watching instructional videos developed in collaboration with physical therapists, followed by demonstrating proper techniques for using both devices. Following the demonstrations, participants were given the opportunity to ask questions and clarify any uncertainties about the transfer procedures. They were provided with time to familiarize themselves with both the PPTS and the mechanical floor lift device. Participants practiced using each device under the guidance and supervision of the study team. They continued practicing transfers until the researchers confirmed they were proficient and ready to proceed with the actual transfer tasks.

#### 2.3.1. Transfers Using Mechanical Lift

**Figure 2:**
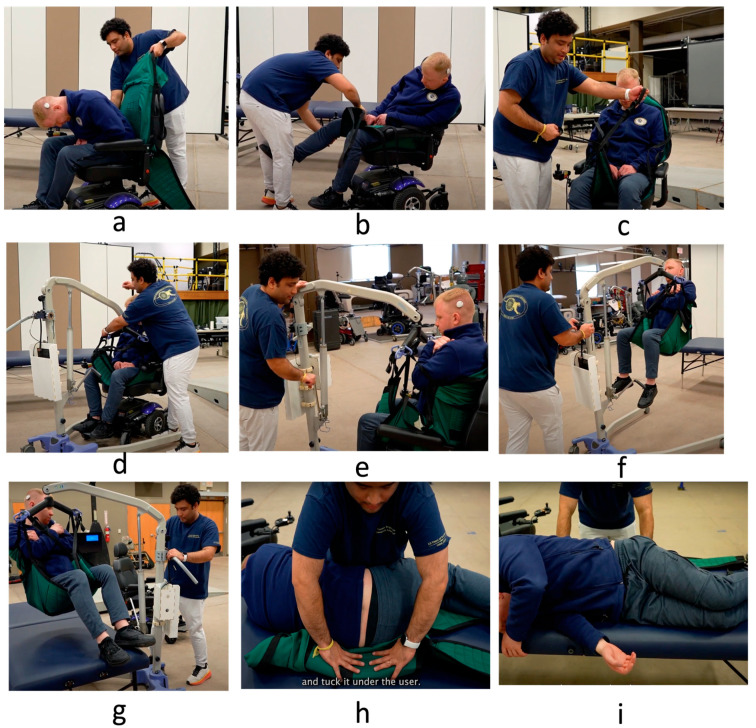
Steps involved (**a**–**i**) in assisting transfers using the mechanical floor lift.

For the mechanical lift transfer, participants were instructed to position the sling behind the wheelchair user’s back (Figure 2a). They were then asked to tuck the sling under the user’s rear. Next, they were asked to lift the user’s legs placing the leg portions of the sling under the user’s thighs (Figure 2b). Participants had to ensure there were no knots or bunching in the sling that could cause discomfort or pressure injuries. After confirming the user’s comfort, participants were instructed to bring the mechanical floor lift close, widen its base using the foot pedal, and securely lock the wheels to prevent movement.

Next, participants were taught to attach the sling straps to the cradle kit of the Hoyer lift, using longer straps for the upper body to reduce chest pressure and medium-length straps for the legs, after crossing the two ends of the sling (Figure 2c,d). They were then shown how to engage the hydraulic lift by turning the knob clockwise and operating the hand crank to lift the user into suspension (Figure 2e). Once the user was suspended (Figure 2f), participants were directed to move the lift toward the bed (or wheelchair) and lower the user gently by turning the knob counterclockwise (Figure 2g) and unhooking the sling strap. Removing the sling involved a multi-step process. Participants were instructed to first lift the legs of the surrogate to remove the foot portion of the sling. Then, they were asked to roll the surrogate onto one side, allowing them to fold and tuck the sling under their body (Figure 2g). After securing the sling in this position, the surrogate was rolled onto the other side, enabling participants to carefully pull the sling out from underneath their body (Figure 2h,i). The participants were instructed to follow similar steps in reverse for transfers from bed to wheelchair.

#### 2.3.2. Transfers Using the PPTS

**Figure 3:**
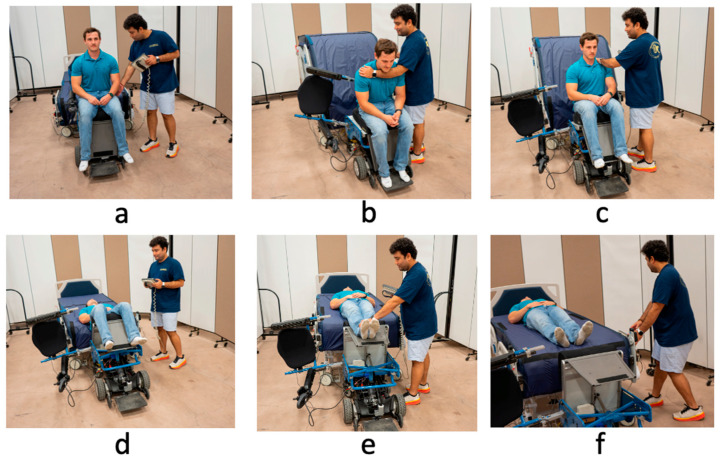
Steps involved (**a**–**f**) in assisting transfers using the PPTS.

Participants were instructed to press the “transfer to bed” option on the touchscreen controller to begin the process (Figure 3a). They were asked to lean the surrogate wheelchair user forward to create enough clearance for the wheelchair’s backrest to swing away as the bed rose simultaneously (Figure 3b). As the seat rotated toward the bed, participants were guided to gently rest the surrogate’s back into the bed with the lower portion of the bed acting as a backrest (Figure 3c). The bed was then lowered, placing the surrogate in a supine position (Figure 3d). At this stage, a conveyor belt over the mattress started rotating toward the headboard while participants continued holding the transfer button on the touchscreen controller (Figure 3e). The bed elevated to ensure a smooth transfer of the surrogate’s feet. Participants were instructed to assist with leg orientation by supporting or repositioning the legs during this phase (Figure 3f). Once the conveyor fully transferred the surrogate’s feet onto the bed, participants were directed to release the “transfer to bed” button, completing the transfer.

### 2.4. Study Protocol

Once the training was completed, participants performed transfers with the surrogate wheelchair user using the PPTS and the mechanical floor lift. The order in which they performed the transfers was counterbalanced across participants to avoid order effects. Each participant completed a total of eight transfers—two transfers from the bed to the wheelchair and two transfers from the wheelchair to the bed using both devices. Throughout the transfer process, inertial measurement units (IMUs) were attached to the participants’ bodies to track their motion during each transfer. A stopwatch was used to measure the time they took to transfer during each trial.

### 2.5. Data Analysis

To compare trunk joint angles between the mechanical floor lift and the PPTS, a MATLAB code was developed for data processing and analysis. The joint angle data collected during the transfer trials were first filtered using a low-pass Butterworth filter of order 4 with a cutoff frequency of 8 Hz to remove high-frequency noise. A fourth-order Butterworth filter with cutoff frequencies between 5 and 10 Hz is common in biomechanical joint data analysis [38,39,40]. For each trial, the maximum and average joint angles for trunk flexion, lateral bending, and axial rotation were calculated using MATLAB version R2024b. Additionally, the time spent in non-neutral postures (trunk angle > 20°) was cumulatively added for the duration of each transfer trial. Statistical analyses were performed using IBM SPSS Statistics software, where Wilcoxon’s rank-sum test was employed to evaluate the hypotheses.

## 3. Results

### 3.1. Participants

Eight participants completed the study (males = 6, females = 2). The mean age ± S.D. was 23.5 ± 6.46 years (range 19–40 years), the mean weight was 72. 74 ± 8.36 Kg (range 65.5–85.72 kg), and the mean height was 177 ± 7.8 cm (range 170–193 cm). None of the participants had previous experience in assisting with transfers.

### 3.2. Time Required to Complete Transfers

As shown in Table 1, transfers using the PPTS were significantly faster compared with the mechanical floor lift. The average transfer time and SD to complete a transfer using the mechanical lift were 525.82 ± 147.19 s. In contrast, the PPTS averaged 144.31 ± 15.94. Transfer times for the mechanical lift ranged from 367.9 to 819.9 s, while the PPTS ranged from 119.3 to 170.2 s. A Wilcoxon signed-rank test showed a statistically significant difference between the two systems (*p* < 0.001), confirming hypothesis 1 that the PPTS enabled significantly faster transfers compared with the mechanical lift. An F-test for differences in variance showed a significant difference between the two systems (F = 85.37, *p* < 0.001), confirming hypothesis 2 that the transfer times with the PPTS were significantly more consistent.

### 3.3. Trunk Joint Angles

We compared the trunk flexion, lateral bend, axial rotation for range of motion (ROM), and average trunk angles during transfers while assisting with transfers using the mechanical floor lift and the PPTS as summarized in Table 1. For trunk flexion, the average range of motion was significantly higher 51.75° ± 11° compared with the PPTS 28.11° ± 8.7° (*p* < 0.001). The average trunk flexion over time was also significantly greater for the mechanical floor lift (mean: 18.73° ± 6.57°) compared with the PPTS (mean: 9.76° ± 5.21°) with *p* < 0.01. In contrast, the lateral bend observed with transfers using the mechanical floor lift showed no statically significant difference compared with the PPTS (mean: 2.44° ± 1.66° vs. 2.94° ± 7.2°). However, the range of motion for lateral bend observed significant differences in the peak values (29.43° ± 10.03° for the mechanical lift vs. 16.95° ± 8.94° for the PPTS, *p* = 0.008). Similarly, the range of motion observed for axial rotation using the mechanical floor lift was significantly greater compared with the PPTS (29.57° ± 12.42° vs. 11.51° ± 6.27°), while the average axial rotation was not statistically significantly different between the two (mean: 4.84° ± 7° vs. 3.34° ± 1.83), *p* = 0.87.

### 3.4. Time Spent in Non-Neutral Trunk Posture

We compared the duration participants spent in non-neutral trunk joint angles, defined as angles exceeding 20°. The time spent with trunk flexion above 20° was significantly higher for the mechanical floor lift (282.16 ± 23.41 s) compared with the PPTS (7.91 ± 8.53 s), *p* < 0.001. Similarly, for lateral bend, the average time spent with lateral bend exceeding 20° was significantly greater for the mechanical floor lift (28.85 ± 23.41 s) compared with the PPTS (2.42 ± 2.79 s), *p* < 0.001. For axial rotation, the average duration spent with axial rotation above 20° was also significantly higher for the mechanical floor lift (35.70 ± 29.38 s) versus the PPTS (4.88 ± 5.18 s), *p* = 0.003.

## 4. Discussion

The results of this study indicated that the PPTS offered significant ergonomic and time benefits over the current standard of care mechanical floor lift when assisting with transfers.

First, in terms of time efficiency, transfers using the PPTS were completed in significantly less time (about 2.4 min) than those with the mechanical floor lift (8.7 min). On average, the PPTS reduced transfer times by approximately 72%, with mechanical lift transfers taking over five minutes longer to complete. This confirmed hypothesis 1, that the PPTS enabled faster transfers compared with the mechanical lift. These findings were consistent with previous studies showing that mechanical lifts, though effective in reducing injury risks due to force exerted on the lower back, are often time-inefficient and require multiple steps for safe operation [16,18]. Additionally, transfers with the PPTS showed significantly less variability, supporting hypothesis 2 that the PPTS enables more consistent transfers, as also evidenced by lowered standard deviations in Table 1. The automation of the PPTS eliminates manual lifting and sling placement, contributing to a faster, more streamlined process that could enhance caregiver efficiency, as well as better adoption in clinical and non-clinical settings.

Regarding trunk joint angles, the PPTS significantly reduced the range of motion during transfers compared with the mechanical floor lift. For trunk flexion, the range of motion required was nearly half as much with the PPTS. Similarly, transfers with the PPTS required significantly lower lateral bend and axial rotation. These findings support hypothesis 3, demonstrating that the PPTS facilitates more ergonomic postures, reducing exposure to non-neutral joint angles that are known risk factors for musculoskeletal injuries [27,28,29]. Specifically, caregivers using the mechanical floor lift experienced significantly higher degrees of trunk flexion, lateral bend, and axial rotation, movements strongly linked to low back pain and discomfort due to increased shear and compressive forces on the spine.

The time spent in non-neutral trunk postures further highlights the ergonomic advantage of the PPTS. Caregivers assisting with transfers using the mechanical floor lift spent significantly more time with trunk flexion above 20° (238.25 s vs. 21.43 s with the PPTS) and lateral bending above 20° (19.49 s vs. 3.78 s). These prolonged periods of awkward posture, especially as seen for trunk flexion, increase the likelihood of injury during repetitive tasks such as transfers. The PPTS significantly reduced the duration of time spent in these known risk postures, aligning with hypothesis 4, which predicted that the system would lower the duration of non-neutral postures, thus reducing the potential for injury. It is also noteworthy that the standard deviations for the time spent in non-neutral postures were large for the mechanical floor lift, emphasizing the variability in transfer techniques among caregivers, even after undergoing identical training.

Admittedly, while the mechanical floor lift is effective in minimizing back forces as per NIOSH criteria when compared with manual transfers, our findings highlight the potential for the PPTS to offer a safer and more time-efficient alternative. Caregivers in long-term care facilities often abandon mechanical lifts due to time inefficiency, despite the increased risk of musculoskeletal (MSK) injuries and pain associated with manual transfers [18]. These injuries, often caused by non-neutral postures and prolonged time spent in such positions, are strongly linked to back pain and MSK disorders [24,27] and can be career-ending, limiting caregivers’ ability to provide long-term assistance. The PPTS addresses these challenges by providing a significantly faster and more ergonomic transfer method, reducing time spent in risky postures, decreasing trunk movement, and minimizing physical strain. By alleviating these physical demands, the PPTS may help mitigate the high incidence of MSK injuries among caregivers, enabling them to maintain their health, extend their careers, and assist more individuals in need.

During the study, we observed several user errors while participants operated the mechanical floor lift, highlighting the complexity of this device. Even with training, participants frequently forgot key steps such as locking the wheels or widening the base of the lift—critical for preventing falls while the user was suspended in the air. Additionally, when lowering the user into the bed or the wheelchair, participants often turned the knob too quickly, leading to rough landings. This abrupt descent could be particularly hazardous for individuals with spinal cord injuries, where even minor impacts might lead to serious health complications. In contrast, the PPTS greatly reduced the risk of such errors due to its automated design and fewer operational steps. Minimal training was required to effectively use the system as its automated functions simplified the transfer process.

In conclusion, the PPTS presents a promising advancement in transfer technologies, offering both ergonomic and time efficiency benefits over mechanical floor lifts. Previous research has highlighted the need for superior transfer technologies to reduce caregiver burden and improve safety during wheelchair transfers [41,42]. Additionally, there is a lack of comprehensive user studies on robotic transfer systems, limiting understanding of their practical benefits. This study addresses both gaps by objectively evaluating the PPTS through direct user studies, providing insights into its efficacy and ergonomic advantages over standard of care methods. However, future studies should include actual wheelchair users to further validate these findings in a clinical context. Additionally, long-term studies are needed to assess the impact of these reduced postures on injury rates and caregiver satisfaction over extended periods of use. Future research should also evaluate factors such as accessibility, cost, and overall usability in real-world settings. Long-term studies evaluating clinical outcomes and transfer efficiency in diverse caregiving environments will provide further insights into its practicality and impact.

## 5. Limitations

In this study, all participants were younger unimpaired individuals with no prior experience assisting with transfers, which may limit the generalizability of the results to trained caregivers. However, it is worth noting that inadequate training in transfer techniques is a well-documented issue among caregivers, often leading to poor posture and inefficient transfers [4,43]. Additionally, participants transferred members of the study team, which ensured consistency and mitigated the risk of physical harm to actual wheelchair users. Nonetheless, the results may differ when the devices are used by or tested with the target population of wheelchair users. While the study offers valuable insights into the ergonomics and time efficiency of transfer systems, including actual wheelchair users and experienced caregivers in future research could provide more clinically relevant findings.

## 6. Conclusions

The study revealed that the PPTS provided notable advantages over traditional mechanical floor lifts in transfer efficiency and ergonomics for transfers between a wheelchair and a bed. Transfers using the PPTS were completed in 2.5 min, approximately 72% faster than with mechanical lifts, confirming its superior time efficiency. Additionally, the PPTS significantly reduced the range of trunk joint angles and time spent in non-neutral postures, addressing ergonomic concerns and potentially lowering the risk of musculoskeletal injuries for caregivers. These promising results pave a pathway for further research involving wheelchair users and caregivers in clinical settings and long-term studies on injury rates and caregiver satisfaction.

## Figures and Tables

**Table 1:** Comparison of time, trunk joint angles, and duration in non-neutral postures during transfers using the mechanical floor lift and PPTS (bold = significant difference).

	Mechanical Floor Lift		PPTS		Wilcoxon’s Signed Rank Test *p*-Values	
	Average	ROM	Average	ROM	Average	ROM
Trunk flexion°	18.73 ± 6.57	51.75 ± 11	9.76 ± 5.21	28.11 ± 8.7	**<0.01**	**<0.001**
Lateral bend°	2.44 ± 1.66	29.43 ± 10.03	2.94 ± 7.2	16.95 ± 8.94	0.47	**0.008**
Axial rotation°	4.84 ± 7	29.57 ± 12.42	3.34 ± 1.83	11.51 ± 6.27	0.87	**0.001**
Transfer time (s)	525.82 ± 147.19		144.31 ± 15.94		**<0.001**	
Duration with flexion > 20° (s)	282.16 ± 23.41		7.91 ± 8.53		**<0.001**	
Duration with lateral bend > 20° (s)	28.85 ± 23.41		2.42 ± 2.79		**<0.001**	
Duration with axial rotation > 20° (s)	35.70 ± 29.38		4.88 ± 5.18		**0.003**	

## Data Availability

All relevant data are contained within the article. To ensure the privacy and confidentiality of the human subjects involved in this project, all other information and data are unavailable due to privacy and ethical considerations.
